# Efforts to improve outcomes among neonates with complex intestinal atresia: a single-center low-income country experience

**DOI:** 10.1007/s00383-024-05639-7

**Published:** 2024-03-06

**Authors:** Innocent Okello, Caroline Q. Stephens, Nasser Kakembo, Phyllis Kisa, Stella Nimanya, Ava Yap, Anne S. Wesonga, Rovine Naluyimbazi, Peter Kayima, Yasin Ssewanyana, Doruk Ozgediz, John Sekabira

**Affiliations:** 1 Mulago National Referral Hospital, Kampala, Uganda; 2 Department of Surgery, Center for Health Equity and Anesthesia, University of California - San Francisco, San Francisco, USA; 3 Makerere University College of Health Sciences, Kampala, Uganda

**Keywords:** Congenital abnormalities, Infant, Newborn, Intestinal atresia, Global health, Disparities

## Abstract

**Purpose:**

Intestinal obstruction caused by intestinal atresia is a surgical emergency in newborns. Outcomes for the jejunal ileal atresia (JIA), the most common subtype of atresia in low-income countries (LIC), are poor. We sought to assess the impact of utilizing the Bishop–Koop (BK) approach to JIA in improving outcomes.

**Methods:**

A retrospective cohort study was performed on children with complex JIA (Type 2–4) treated at our national referral hospital from 1/2018 to 12/2022. BK was regularly used starting 1/1/2021, and outcomes between 1/2021 and 12/2022 were compared to those between 1/2018 and 12/2020. Statistical significance was set at *p* < 0.05.

**Results:**

A total of 122 neonates presented with JIA in 1/2018–12/2022, 83 of whom were treated for complex JIA. A significant decrease (*p* = 0.03) was noted in patient mortality in 2021 and 2022 (*n* = 33, 45.5% mortality) compared to 2018–2020 (*n* = 35, 71.4% mortality). This translated to a risk reduction of 0.64 (95% CI 0.41–0.98) with the increased use of BK.

**Conclusion:**

Increased use of BK anastomoses with early enteral nutrition and decreased use of primary anastomosis improves outcomes for neonates with severe JIA in LIC settings. Implementing this surgical approach in LICs may help address the disparities in outcomes for children with JIA.

**Supplementary Information:**

The online version contains supplementary material available at 10.1007/s00383-024-05639-7.

## Background

Among newborns, intestinal obstruction is a common surgical emergency, often caused by intestinal atresia [1, 2]. Jejunal ileal atresia (JIA) is the most common subtype, with an annual incidence from 0.7 to 30 in 10,000 live births [2–4]. While outcomes in neonatal surgery have significantly improved in high-income countries (HIC), this is not the case in low-income countries (LIC) [5, 6]. A multinational study involving 74 centers found that the mortality in LICs was 60% among patients with intestinal atresia compared to 3.3% in HIC settings.[5]. In our center, we previously found that mortality for all types of bowel atresia was 45%, with JIA accounting for the largest proportion of these deaths [7, 8]. The reasons for these disparities are multifactorial, as delayed diagnosis, lack of infrastructure to facilitate patient transfer, lack of neonatal intensive care, and lack of parenteral nutrition are common in LIC settings. These resource limitations compound one another due to the critical role that nutrition plays in neonatal survival. Thus, attention to the available nutritional options is crucial when considering the surgical treatment of JIA neonates.

Multiple surgical techniques can be employed to manage JIA, each with advantages and drawbacks. While resection of the atretic segment and primary anastomosis spares the patient from an ostomy, the risk of anastomotic leak and delayed return of bowel function can be deadly without total parenteral nutrition (TPN). Creating a diverting stoma is a viable alternative, but creates the potential for high stoma output and malabsorption. An ostomy in continuity, such as the Bishop–Koop (BK) technique, the Santuli enterostomy, or the Mikulicz procedure, is an attractive option in low-resource settings [9]. These techniques offer the benefits of ostomy refeeding and re-establishment of continuity, reducing the extent of the subsequent ostomy closure [10]. In some studies, the Bishop–Koop procedure has shown fewer surgical complications, shorter hospital stays, and less operating time [10].

Given the above evidence and the high mortality noted in the Ugandan setting, [8, 10, 11], a small number of individual surgeons at our tertiary care referral center decided to utilize this approach for complex JIA (Type 2–4) starting in 2019. In addition, in 2020, a process for the inclusion of amino acids and dextrose in the intravenous fluids for all infants with gastrointestinal congenital anomalies was adopted. As a result of improved anecdotal outcomes, BK was adopted in 2021 by the entire surgical group as the standard procedure for patients with complex JIA. This was accompanied by the creation of a management process to maximize early enteral nutrition (EEN) for JIA neonates through the administration of feeds through the ostomy created by the BK approach. We sought to compare the outcomes of children with JIA before and after the standard use of BK procedure for the care of complex JIA for neonates in Uganda.

## Methods

### Study design

We conducted a retrospective cohort study of neonates with complex JIA at our tertiary care referral center in Kampala, Uganda. All infants with complex JIA who underwent surgical treatment between January 1, 2018 and December 31, 2022 were included in our study. Outcomes for neonates who underwent surgical intervention following January 1, 2021 were compared to those who underwent intervention between 1/1/2018 and 12/31/2020. IRB approval was obtained from our hospital IRB (MHREC #464).

### Study participants

Complex JIA was defined as any neonate with Type 2, Type 3, or Type 4 JIA. Any neonate with a diagnosis of Type 1 JIA or who had an undesignated type but underwent a web excision, the standard surgical approach to Type 1 JIA, was excluded from our study. We also excluded all neonates with an undesignated JIA type and unspecified surgical procedure. All those with concomitant gastrointestinal anomalies, including duodenal atresia, colonic atresia, or gastroschisis, were excluded. Lastly, any neonate with suspected JIA who expired before surgical intervention was excluded.

### Complex JIA care pathway

**Fig. 1:**
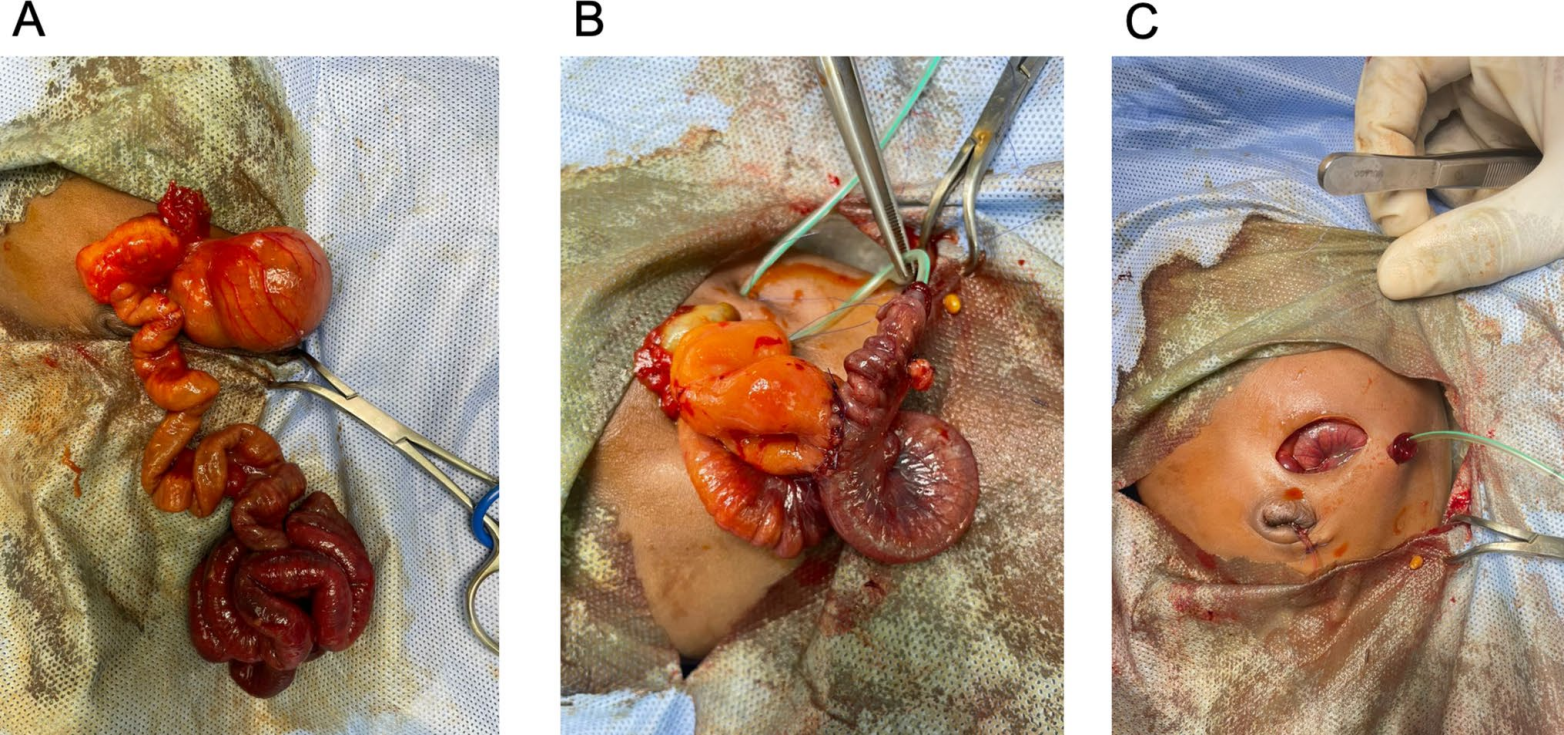
Creation of Bishop–Koop (ostomy-in-continuity) anastomosis. Exploratory laparotomy is performed, and the neonate is found to have a Type 3B jejunoileal atresia (**A**). An end-to side anastomosis is created between the dilated proximal intestine and narrow distal intestine, and a feeding tube is passed through the distal limb and brought out at the planned ostomy site (**B**). Ostomy-in-continuity is created with feeding tube in place (**C**)

Between 2018 and 2020, the typical approach utilized to treat patients with complex JIA was either a primary anastomosis or ostomy creation, with a small number of surgeons using the BK approach. However, at the end of 2020, the pediatric surgery team decided collectively to utilize the BK ostomy-in-continuity approach regularly. This method results in an end-to-side anastomosis between the dilated proximal intestine (end) and small distal intestine (side), with the end of the distal intestine brought out to make a single ostomy. A size 5 French feeding tube was also passed down the distal intestine to facilitate refeeding of nasogastric tube aspirates as well as breast milk (Fig. 1).

Following the observation of improved anecdotal outcomes from individual BK cases, the decision was made at the end of 2020 to adopt BK as the standard approach used in the treatment of complex JIA. This was accompanied by a change in management of JIA neonates to support EEN, which included utilization of the feeding tube down the distal intestine to facilitate refeeds (Supplement 1). These distal feeds include both the nasogastric tube output that is aspirated, as well as expressed breast milk at 2 mL every 2 h starting 24 h from surgery. Additionally, as TPN was unavailable, amino acids and dextrose were added to intravenous fluids to support nutritional needs while awaiting the return of bowel function. The inclusion of amino acids and dextrose in intravenous fluids for neonates at risk for delayed return of bowel function was initiated at the beginning of 2020.

### Data sources and variables

Clinical data were obtained from our prospectively collected inpatient database of all pediatric surgery patients maintained at our hospital. This database was created in 2012, and all epidemiologic and outcome data of pediatric surgical patients were collected by a non-clinical staff hired by the pediatric surgical team. This database consistently includes data on patient presentation, diagnosis, and surgical interventions. A free text section also exists to include specifics on the type of operation performed, abstracted from operative logs. Outcomes included were death, survival to discharge, and left against medical advice. All data were abstracted from the paper patient charts. Due to the breadth of patients included and available staff for data entry, the database does not include data on specific interventions, such as the dates of enteral feed initiation and the volume of feeds administered.

Epidemiologic variables, including patient demographics, presenting complaints, and atresia type, were examined. To understand the success of our protocol implementation, we examined different operative approaches over time, specifically focusing on primary anastomosis versus BK. Our primary outcome was the percent mortality per year; the secondary outcome was the patient's age at death. The lack of data on enteral feed initiation, volume, and rate limited any analysis of the specific change caused by the EEN protocol.

### Statistical analysis

All data analyses were performed using STATA 17. Epidemiologic data were evaluated using Chi-square and Fisher’s exact tests, as appropriate. The Cochran–Armitage test for trend was used to examine the change in operative approach over time. A risk ratio for mortality was calculated before and after standard BK use. Mann–Whitney *U* tests were used to compare age at death for all those neonates who died. *p* value < 0.05 was considered significant.

## Results

### Participants

**Fig. 2:**
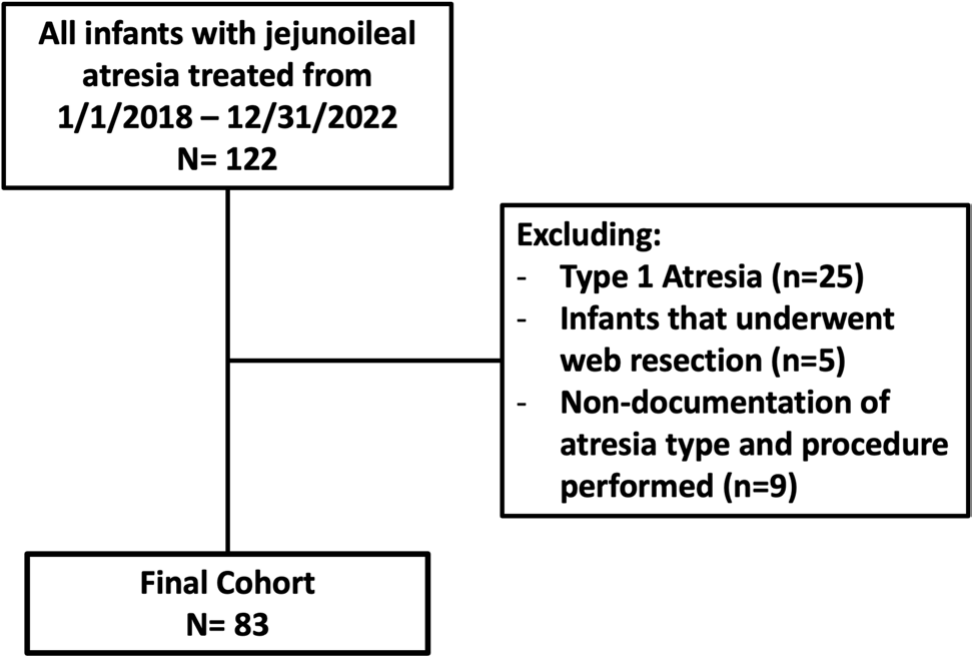
Flow diagram of cohort creation for the study

In total, 122 neonates presented with JIA between 1/1/2018 and 12/31/2022. Of these, 25 patients with Type 1 JIA and 5 patients who underwent web resections were excluded from the study. We also excluded an additional nine patients who neither had documentation of the atresia type nor a specified surgical approach (BK, ostomy, or primary anastomosis) to ensure that only those patients who would have been candidates for BK were included in the study (Fig. 2). While many patients did not have documentation of the atresia type, they had a specified non-web excision surgical approach. As such, they were presumed to be BK candidates and were included in the study (Table 1). This resulted in a final cohort of 83 patients who underwent surgical treatment of complex JIA; 43 were treated from 2018 to 2020, and 40 were treated between 2021 and 2022.

**Table 1:** Distribution of atresia type within study cohort

	Total *N* = 83		2018–2020 *N* = 43		2021–2022 *N* = 40	
	*N*	%	*N*	%	*N*	%
Type 2	4	4.8	2	4.7	2	5.0
Type 3	20	24.1	4	9.3	16	40.0
Type 4	24	28.9	15	34.9	9	22.5
Unknown	35	42.2	22	51.2	15	32.5

### Demographics

Overall, the two cohorts had no significant differences in the demographic data. For both groups, the median age at presentation was 7 days, and most neonates were referred from local health centers within 50 km of our tertiary referral center (Table 2). Over 60% of patients lived in one of the two districts around the capital city (Kampala or Wakiso). Most patients presented with complaints of vomiting with or without abdominal distension or failure to pass meconium.

**Table 2:** Presenting characteristics for neonates with jejunoileal atresia

	Total *N* = 83		2018–2020 *N* = 43		2021–2022 *N* = 40		*p*-value
	*N*		*N*		*N*		
*Age of presentation (M, IQR)*	78	7 (4–11)	40	7 (4.5–9)	38	7 (4–13)	0.6
*Sex (% male)*							0.22
Male	37	44.6%	16	37.2%	21	52.5%	
Female	42	50.6%	24	55.8%	18	45.0%	
Missing	4	4.8%	3	7.0%	1	2.5%	
*Distance traveled (%)*							0.2
< 50 km	50	76.9%	29	85.3%	21	67.7%	
50–100 km	4	6.2%	2	5.9%	2	6.5%	
> 100 km	11	16.9%	3	8.8%	8	25.8%	
Missing	18	21.7%	9	20.9%	9	22.5%	
*Presenting complaints (%)*							0.18
Vomiting	19	22.9%	13	30.2%	6	15.0%	
Vomiting and lack of meconium passage	10	12.1%	2	4.7%	8	20.0%	
Vomiting, distension, and lack of meconium passage	6	7.2%	3	7.0%	3	7.5%	
Abdominal distension	3	3.6%	1	2.3%	2	5.0%	
Lack of meconium passage	3	3.6%	2	4.7%	1	2.5%	
Vomiting and distension	5	6.0%	4	9.3%	1	2.5%	
Distension and lack of meconium passage	4	4.8%	2	4.7%	2	5.0%	
Other	1	1.2%	0	–	1	2.5%	
Missing	32	38.6%	16	37.2%	16	40.0%	

### Change in surgical approach over time

**Fig. 3:**
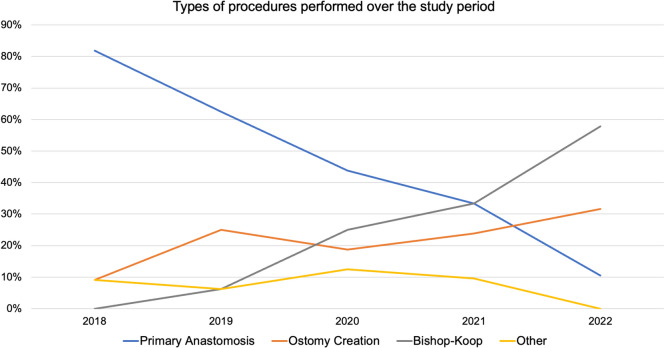
Changes in the types of procedures performed on infants with JIA over the study period

The annual proportion of BK procedures performed rose from 0% (*n* = 0) to 58% (*n* = 11) over the study period, while the proportion of primary anastomoses performed fell from 81.8% (*n* = 9) to 10.5% (*n* = 2). A significant change was observed in the types of procedures performed over time. Specifically, a trend toward decreased use of primary anastomosis (*p* < 0.001) and increased use of the BK approach (*p* < 0.001) was observed (Fig. 3). No significant difference was found in the use of ostomy creation (*p* = 0.23) or other unspecified procedure (*p* = 0.52).

### Patient outcomes

In comparing the cohorts, a significant decrease (*p* = 0.03) was noted in patient mortality in 2021 and 2022 (*n* = 33, 45.5% mortality) compared to 2018–2020 (*n* = 35, 71.4% mortality). This translated to a risk reduction of 0.64 (95% CI 0.41–0.98) with increased BK use and implementation of EEN with ostomy refeeding. While not significant (*p* = 0.10), the median age of death in 2021–2022 was 21 days (IQR 14–24) compared to 14 days (IQR 10–21) in 2018–2020.

## Discussion

This is the first low-income country (LIC) study to examine the outcomes of neonates with JIA who undergo surgical repair with the BK technique versus the standard primary bowel anastomosis. Investigation of these approaches in the LIC setting is critical, as the availability of TPN in LIC institutions is 19% versus 93% in middle-income countries and 100% in HIC settings [12]. Thus, neonates in LICs primarily rely on early return of bowel function for survival, and surgical approaches must adapt to maximize survival potential and nutrition, as was explored in this study.

We found a significant decrease in mortality among neonates with JIA following the standard use of the BK operative approach and implementation of ostomy refeeding with EEN. Additionally, while not significant, an increased inpatient life span was noted in patients with complex JIA who died, from 2 to 3 weeks. These outcomes suggest that combining a change in surgical approach with a focus on maximizing nutrition for complex JIA can significantly reduce neonatal mortality from this congenital anomaly in LICs. In particular, the use of an ostomy-in-continuity approach rather than primary anastomosis facilitates the administration of EEN by providing an option for ostomy refeeding. In settings where TPN is lacking, consideration of surgical approaches that facilitate nutrition is critical for improving outcomes.

These findings corroborate prior studies examining BK use for JIA and is one of two comparative studies in settings with limited TPN access. These previous studies demonstrated decreased mortality for neonates with JIA who undergo BK: 22% (*n* = 9) in Bangladesh (no TPN access), 7.3% (*n* = 41) in China (2018, TPN access), 7.1% (*n* = 41) in China (2019, TPN access), and 13% (*n* = 30) in India (mixed fluid used as a replacement for TPN) [9, 11, 13, 14]. Due to the variance in TPN access, it is difficult to draw direct comparisons from these studies. However, a similar trend is noted with decreased complications among those children who undergo ostomy-in-continuity vs primary anastomosis [9, 14]. In particular, in the setting that used mixed fluids as a replacement for TPN, the mortality was 33% in the primary anastomosis (PA) group compared to 13% in the BK group [9]. In the mixed fluids study, PA patients also had the highest reoperation rate (27%), with 20% of PA patients experiencing an anastomotic leak [9]. Thus, our study corroborates that BK has superior outcomes to PA in settings with limited access to TPN. In addition, while not collected in our research, prior works from China and India suggest that the BK approach also lowers the frequency of serious postoperative complications (anastomotic leak, intestinal obstruction, reoperation) [9, 14].

Several factors increase the risk of complications in patients with severe JIA following surgical repair. In particular, the size mismatch between the proximal and distal bowel can result in ineffective peristalsis, and the blood supply to the distal limb can be tenuous. These factors place stress on the anastomosis, especially in the setting of malnutrition, which may lead to higher rates of anastomotic breakdown. The ensuing sepsis from leakage may also place the malnourished neonate at a higher risk of death. It was due to these factors that the BK approach was considered for use in the Ugandan setting, given the prior evidence suggesting leakage rates were lower, as well as the potential for providing EEN through the distal intestine earlier in the postoperative course. However, since the practice change was implemented, two additional studies have explored using multiple types of ostomy-in-continuity for JIA. One study compared PA (*n* = 49) versus BK (*n* = 42) versus Mikulicz double-barreled ileostomy (*n* = 14) and found that both BK and Mikulicz had reduced complications. While BK had three (7%) anastomotic leaks, three (7%) perioperative deaths, and two (5%) reoperations, Mikulicz had nine (64%) high-output stomas, two (14%) reoperations, no leaks, and no deaths [14]. Similarly, in a separate study that compared PA (*n* = 30), BK (*n* = 30), Santulli (*n* = 27), and Mikulicz (*n* = 25) for JIA, BK was noted to have high rates of anastomotic leaks (10%) as compared to Santulli (3.7%) or Mikulicz (0%). [9] However, conversely, high-output stomas were the least common for those who underwent BK (3.3%) versus Santulli (22%) and Mikulicz (8%) [9]. Overall, given that in both settings, a method of parenteral nutrition was employed, either via formal TPN or a mixed fluid, it is difficult to know from these data how complications may have differed in an LIC setting with no TPN. Even so, while these studies corroborate the improved outcomes when ostomy-in-continuity is used over PA, they also raise the question of the appropriate operative approach in low-resource settings. In these settings, it is critical to consider the risk of anastomotic breakdown and the child dying from sepsis versus the risk of a high-output stoma with the child succumbing to malnutrition. As the sample sizes in these studies are small, further work is needed, especially in LIC settings, to better assess the optimal approach to neonates with severe JIA.

The high rate of mortality observed in our study is similar to the mortality for intestinal atresia observed in multiple other studies [5, 7, 15]. This supports the generalizability to LIC settings, especially as this study is one of the largest to examine outcomes among children with complex JIA. Many of these prior studies had smaller sample sizes, ranging from 20 to 40 patients. The high mortality noted among JIA patients, higher than all other types of atresia, is multifactorial due to a combination of delayed diagnosis, lack of neonatal intensive care resources, and lack of options for parenteral nutrition [4–6]. The median age of presentation of 7 days likely directly contributed to the mortality observed in our study. Our hospital is the only national referral center for pediatric surgical conditions with a catchment area that spans the country and neighboring countries. Once the family recognizes that the child is not doing well, they must present to their local center for care, receive a diagnosis of possible atresia, and then travel, often on boda-boda (a type of motorcycle), hundreds of kilometers for care. Many patients present in a dehydrated, cold, and malnourished condition with or without sepsis, placing them at further risk of complications. Late diagnosis compounds the challenging nutritional outcomes, as surgical interventions may occur after a week of life. As a result, the child must survive for multiple weeks without enteral or parenteral nutrition. Thus, the use of surgical techniques that maximize nutrition is critical to improving survival.

Several interventions were utilized to address the challenges posed by the lack of access to TPN for our patient population. This included the addition of amino acids to intravenous fluids and using fresh frozen plasma transfusions to support neonates with hypoalbuminemia. Additionally, the ward adopted a process for facilitating EEN through ostomy refeeding, as described in Supplement 1. This included refeeding of both nasogastric tube aspirates and expressed breast milk starting 24 h from surgery through the ostomy feeding tube. In addition, an effort was made to start nasogastric and oral enteral feeds as early as possible. Due to the limitations of the pediatric ward database, it is difficult to assess the impact of these interventions. However, the significant improvement in mortality seen in 2021 and 2022 is likely facilitated by the improvement in nutrition for these neonates. In addition, the increase in the median length of life by 1 week in the 2021–2022 cohort for those neonates who did not survive was likely facilitated by the improvement in nutrition. The approach described in the supplement could also be reproducible in similar settings with similar resource constraints.

There are several uncontrolled confounding variables in our study design. Due to the lack of an electronic medical record and the limited data collected by the inpatient registry, it was not possible to determine which children may have presented with sepsis, aspiration pneumonia, or under-resuscitation. However, it would be expected that these confounding factors would have made children more likely to expire rather than less likely. It is reassuring that a change between the two cohorts was observed despite the lack of control for these factors. Limited information was also available on postoperative complications such as anastomotic leak, intestinal obstruction, malnutrition, high ostomy output, or sepsis. As a result, the difference in anastomotic leak rate between the primary anastomosis cohort and the BK cohort could not be assessed. However, as improved outcomes were noted as primary anastomosis use decreased, this likely represents a decrease in postoperative complications associated with the surgical approach.

This study has several limitations. First, this represents a single-center experience, and the cohort size is small owing to the rarity of this disease. This may limit the generalizability of our findings. However, this work is the third study to demonstrate improved outcomes using the ostomy-in-continuity approach in low- and middle-income countries and the first in the LIC setting. These studies together suggest that a change away from using primary anastomoses for severe JIA is needed in these settings, and future multi-center studies may be warranted to determine the best approach.

Our study analysis was also limited by missingness as the type of JIA was not always recorded, and some procedure names were non-specific, such as “exploratory laparotomy.” As described in the methods, an effort was made to remove all Type 1 atresia from this analysis by removing any child listed as having Type 1 atresia, any child who had a web resection, which is only performed on children with Type 1 atresia, and by removing all children who had non-specified procedures and non-specified diagnoses. This allowed for the creation of a single cohort with Type 2–4 atresia. However, the missingness in procedure specification did limit the direct comparison of PA versus BK. The study team was unsure whether the “ostomy creation” procedures were BK procedures, as this approach includes ostomy creation. To address this challenge and as the entire team of pediatric surgical faculty collectively decided to utilize BK in 2021, the cohorts were assessed based on the time frame during which they received care. This decision is supported by the significant decrease in PA use during the study period. This change and the significant increase in BK use support the collective standardization of the surgical approach to care for JIA neonates. However, this method of analyzing the patient outcomes likely introduces uncontrolled confounding. In addition, it was also impossible to assess the impact and success of the EEN protocol in starting feeds earlier due to the lack of data on feeding in our database. Future assessment of the outcomes of JIA neonates with the consistent collection of operative approach, preoperative risk factors, and postoperative complications is needed.

## Conclusion

Overall, this study supports using Bishop–Koop anastomoses with a focus on EEN for neonates with severe JIA in LICs. As corroborated by our study, using primary anastomoses for severe JIA in low-resource settings is associated with increased postoperative complications and mortality. Future multi-center studies assessing the different ostomy-in-continuity approaches to severe JIA are sorely needed and could help to improve outcomes for these vulnerable children across the globe.

## Supplementary Information

Below is the link to the electronic supplementary material.

Supplementary file1 (DOCX 16 KB)

## Abbreviations

BK: Bishop Koop
EEN: Early enteral nutrition
PA: Primary anastomosis
HIC: High-income country
JIA: Jejunoileal atresia
NICU: Neonatal intensive care unit
LIC: Low-income country
TPN: Total parenteral nutrition
