# Supplementary material for: Efforts to improve outcomes among neonates with complex intestinal atresia: a single-center low-income country experience
Source: Pediatr Surg Int. 2024 Mar 6;40(1):70. doi: 10.1007/s00383-024-05639-7 (PMC10917857; doi:10.1007/s00383-024-05639-7)
Supplement: Supplementary file 1 — Supplementary file1 (DOCX 16 KB) [file 383_2024_5639_MOESM1_ESM.docx]

Supplement 1. **Mulago Pediatric Surgery Unit Jejunal Atresia Management using Bishop Koop**

1. Do all the necessary investigations to make the diagnosis and for presurgical operation.
2. Place nasogastric tube (NGT) and establish intravenous access. NGT actively aspirated and all loses are replaced with an equal volume of crystalloid.
3. Determine fluid status and resuscitate with fluid blouses at 20mLs/kg to correct dehydration. If baby is well hydrated, then maintenance fluids should be calculated per kg body weight. Fluids should be given as a cocktail of 5% amino acids and 10% dextrose solution (The 10% dextrose solution is mixed on the unit by the surgical team combining 50% dextrose with the available crystalloid, preferable ringers lactate).
4. Maintain warmth of the neonate during resuscitation and exposure.
5. Intraoperation; Bishop koop procedure is done through a supraumbilical transverse laparotomy incision. The 5 french feeding tube is inserted in the distal bowel limb from a skin incision where the stoma will be placed prior to completion of the end-to-side anastomosis. The anastomosis is then completed and the distal bowel limb exteriorized as a stoma.
6. Immediate post operation, the maintenance intravenous fluids and aminoacids (AA) are continued.
7. Twenty-four hours after the operation, 10 mL of room temperature normal saline is pushed through the feeding tube in the distal bowel limb.
8. Feeding is started with Expressed Breast Milk (EBM) at 2mls every two hours through the feeding tube in the distal bowel limb.
9. Keep the NGT on 2 hourly aspirations (train the caretaker in how to do this and to record output).
10. The NGT aspirates are fed into the feeding tube in the distal bowel limb every time they are withdrawn from the stomach. If at any time the infant demonstrates abdominal distension or intolerance of these distal feeds, these feeds are stopped until distension is investigated and reduced.
11. Initiate broad spectrum intravenous antibiotics and proton pump inhibitors. Post operation analgesia is also administered.
12. Obtain postoperative laboratory tests (CBC, serum electrolytes, albumin). If deranged, these are corrected accordingly, with repeat labs checked as needed.
13. When the NGT aspirate volumes are less than 2mLs over 2 hours, the 2 hourly aspirations stop. Then EBM is then given through the NGT and the feeding tube in the distal bowel making a total according to the baby’s daily fluid intake requirements, including the intravenous fluids and AA.
14. As enteral feeds go up, adjust the intravenous maintenance fluids to maintain the total fluid volume as per the weight of the neonate.
15. Once a total volume of 10mls EBM 2 hourly has been achieved, the neonate is allowed to breastfeed.
16. EBM is increased in 2ml increments daily as the IV fluids and AA are reduced accordingly.
